# A dataset for multi-faceted analysis of electric vehicle charging transactions

**DOI:** 10.1038/s41597-024-02942-9

**Published:** 2024-03-01

**Authors:** Keon Baek, Eunjung Lee, Jinho Kim

**Affiliations:** 1 Department of Electrical Engineering, Chosun University, Gwangju, 61452 Republic of Korea; 2 Research Institute for Solar and Sustainable Energies, Gwangju Institute of Science and Technology, Gwangju, 61005 Republic of Korea; 3 School of Energy Convergence, Gwangju Institute of Science and Technology, Gwangju, 61005 Republic of Korea

**Keywords:** Energy economics, Energy and behaviour, Energy management

## Abstract

This study discloses a dataset of electric vehicles’ (EVs’) charging transactions at a scale for multi-faceted analysis from both EV charger and user perspectives. The data comprises whole sessions that occurred during a charging operation company’s annual commercial operation period, specifically including identifiers and charger location categories. For data acquisition, machine-to-machine wireless communication system with proper retransmission for interruption is utilised. The entire dataset is newly collected and is available with 72,856 sessions from 2,337 EV users and 2,119 chargers. The dataset can be used in a variety of ways for the functioning of power systems and markets, including EV charging service businesses, charger installation siting, demand transaction market design, and long-term investment planning of EV-related infrastructure.

## Background & Summary

To overcome climate change and achieve carbon neutrality, the transportation sector is undergoing an energy load shift, and there have been discussions and proposals in many countries to phase out the production and use of internal combustion engines over the next few decades. For example, targets have been set in Norway to ban new sales of petrol and diesel vehicles by 2025, in the UK by 2030, and in California by 2035, and major vehicle manufacturers have announced plans to eliminate the internal combustion engine and introduce electric or other alternative powertrains in the coming years^1,2^. In addition, in the EU, all new passenger and commercial cars registered in Europe must achieve zero emission by 2035. As an intermediate step, ‘Fit for 55’, which refers to the reductions of 55% and 50% in carbon emissions for new cars and vans respectively by 2030, was adopted^3,4^.

Under international consensus, manufacturing systems are being modified to increase the production of electric vehicles (EVs) and sales prices are being reduced to encourage the uptake of EVs without relying on promotions^5,6^. EV chargers are also being developed and demonstrated to provide services related to power system operations, such as power reserve and volatility response^7–9^. Charging rates and service designs are being considered to reflect the price sensitivity of EV users and the transaction cost characteristics of renewable energy in wholesale electricity markets^10–12^.

On the other hand, there are several concerns within the aforementioned industry sentiment. Firstly, the disproportionate proliferation of EVs dramatically increases the electricity demand on the distributed power system. The power consumed to charge a single EV is comparable to the power consumption of 20 conventional households, and by 2030, the power system energy will be increased globally by 525–860TWh following expected EV penetration rates^13,14^. In particular, without an analysis of the charging behaviours of EVs, not only is it difficult to utilise them as a demand resource to respond to the variability of renewable energy, but it also adds to the uncertainty of system net load forecasts^13,15^. Unfortunately, in previous studies, data obtained from just a small number of volunteers under experimental conditions were publicly available with only limited information, as shown in Table 1^16–21^.

**Table 1:** Summary of the details in public EV charging datasets.

Dataset	The number of sessions	The number of EV	The number of charging stations	Resolution	Duration
ACN-Data^16^	+30000	—	54	—	—
Energy consumption of 15 electric vehicles^17^	5490	15	—	1 day	1 year
Replication Data for: A Field Experiment on Workplace Norms and Electric Vehicle Charging Etiquette^18^	3395	85	105(site: 25)	1 second	2014.11.15.~2015.10.01.
One year recordings of electric vehicle charging fleet^19^	—	10	30	10 minutes	2018.05.11.~2020.07.01.
Electric Vehicle (EV) and EV Charging Station Data^20^	16505	—	524	—	—
Electric Vehicle Charging Transactions^21^	5486	—	82	1 second	2021.04.01.~2022.04.01.

Furthermore, data-driven behavioural characterisation is required for decision-making and coordination of stakeholders such as manufacturers, charging operators, and power system operators involved in the economic planning of EV and charger proliferation. At the same time, there is a need to improve social awareness of EV user inconveniences^22–26^. Although methods to generate meaningful data have been studied as shown in Table 2^27–35^, the data generated through experiments have clear limitations for an empirical behavioural analysis.

**Table 2:** Summary of synthetic data generation methods for EV data.

Approach	Type of data
Generative adversarial network(GAN)^27^	EV arrivals, Departure time
Markov chain model^28^	EV arrivals
Beta mixture model^29^	EV arrivals, Departure time
Gaussian mixture models(GMMs)^30^	EV arrivals
Uni/multi-modal data distributions^31^	Departure time
Conditional probability distribution^32,33^	Departure time
K-nearest neighbor algorithm^34^	Charging demand
Auto-regressive algorithm^35^	Charging demand

Therefore, in this paper, the authors present a dataset of charging sessions that is large enough to allow for analysis from both the EV charger and user perspectives, specifically including identification information and location categories. To account for seasonality, the data consists of sessions that occurred during the annual commercial operation period. Accordingly, the resulting dataset is a unique and valuable consideration in several analyses, including

- Service businesses based on customer inconvenience estimates and forecasts and cost-effectiveness calculations, including time-varying charging rates and reservation services
- Charger installation siting design based on analyses of charger location information
- Design of power demand transaction markets, programmes, tariffs, and incentives to promote load shifts and respond to renewable energy variability
- Long-term investment planning for infrastructure, including distribution grids and charging stations

## Methods

### Data collection process

**Fig. 1:**
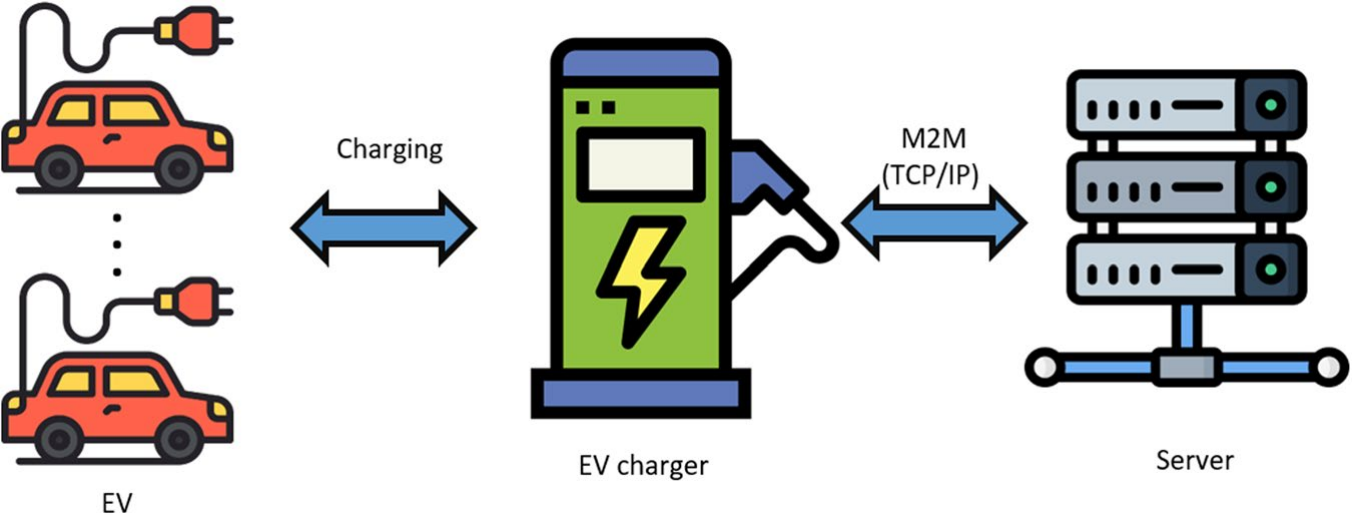
Overall hardware communication network.

**Fig. 2:**
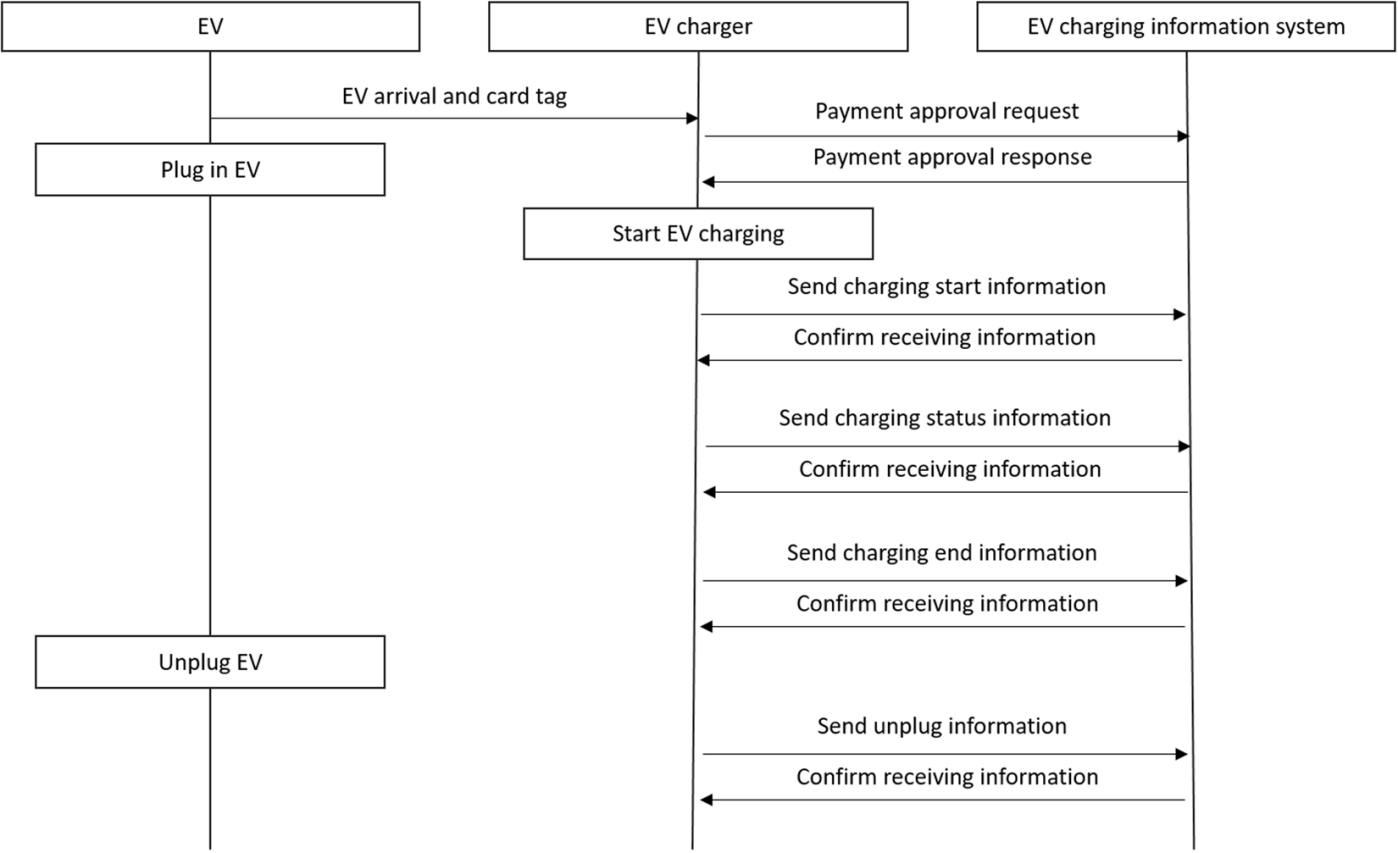
Example of the main operation after the EV couples with the charger.

The line between the charging information system and the charger uses TCP/IP based on machine-to-machine (M2M) wireless communication as shown in Fig. 1. The communication protocol follows the open charge point protocol (OCPP), an industry standard developed for the purpose of operation and maintenance of charging stations in the open charge alliance. The user is identified by entering the RF card tag or unique ID number held by the user. The charger transmits information (membership card tag, charger connection, charging status, charger disconnection, charging error, etc.) acquired in real time to the charging information system at a frequency of 30 seconds. Large data such as firmware installation files and membership information are transmitted to the charger via file transfer protocol (FTP). The communication port is opened and closed according to the time-out interval of the packet. In general, the communication process of sending request and receiving response is configured in a 30-second cycle. In case of communication failures, the process is repeated twice in 15-second cycles, and the charging history and alarm data generated during the communication interruption are separately transmitted to the charging information system through a retransmission-only packet. Figure 2 describes an example of the main operation after the EV couples with the charger and the data communication process during the cycle.

### Coupling status estimation between EV and charger

In this study, the vehicle-charger coupling profile of each session is estimated based on the charging start and end times. The time detection information within sessions are converted into daily time-series data with a resolution of 15 minutes, and the data are classified for analysis purposes using the date, location, and de-identified EV and charger information.

### Responsiveness to EV charging rates

To extend the utility of the proposed data, the authors present the EV charging tariffs imposed on EV users and charging operators in this study in Tables 3, 4^36^.

**Table 3:** Summary of EV charging tariff in South Korea.

Classification		Demand charge (KRW/kW)	Energy charge (KRW/kWh)			
			Time period	Summer	Spring/fall	Winter
Low-voltage	Option 1	2390	Off-peak	87.9	77.4	102.6
			Mid-peak	154.2	89.2	135.1
			On-peak	195.5	94.1	164.0
	Option 2	2390	Off-peak	75.1	77.4	97.8
			Mid-peak	132.0	89.2	118.7
			On-peak	262.8	94.1	219.0
	Option 3	2390	Off-peak	82.1	77.4	107.5
			Mid-peak	130.6	89.2	117.4
			On-peak	228.0	94.1	190.4
	Option 4	2390	Whole time	164.0	89.2	146.9
High-voltage	Option 1	2580	Off-peak	81.8	72.2	91.4
			Mid-peak	121.9	83.0	110.4
			On-peak	143.2	86.9	124.4
	Option 2	2580	Off-peak	70.2	72.2	87.2
			Mid-peak	105.0	83.0	97.5
			On-peak	190.6	86.9	164.4
	Option 3	2580	Off-peak	76.5	72.2	95.6
			Mid-peak	103.9	83.0	96.5
			On-peak	166.0	86.9	143.6
	Option 4	2580	Whole time	129.4	83.0	119.7

**Table 4:** Season and time-period classification for EV charging tariff.

Classification	Summer (6~8)	Spring/fall (3~5/9~10)	Winter (11~2)
Off-peak	22:00~08:00	22:00~08:00	22:00~08:00
Mid-peak	08:00~11:00	8:00~11:00	8:00~09:00
	12:00~13:00	12:00~13:00	12:00~16:00
	18:00~22:00	18:00~22:00	19:00~22:00
On-peak	11:00~12:00	11:00~12:00	09:00~12:00
	13:00~18:00	13:00~18:00	16:00~19:00

### Plus DR program

**Fig. 3:**
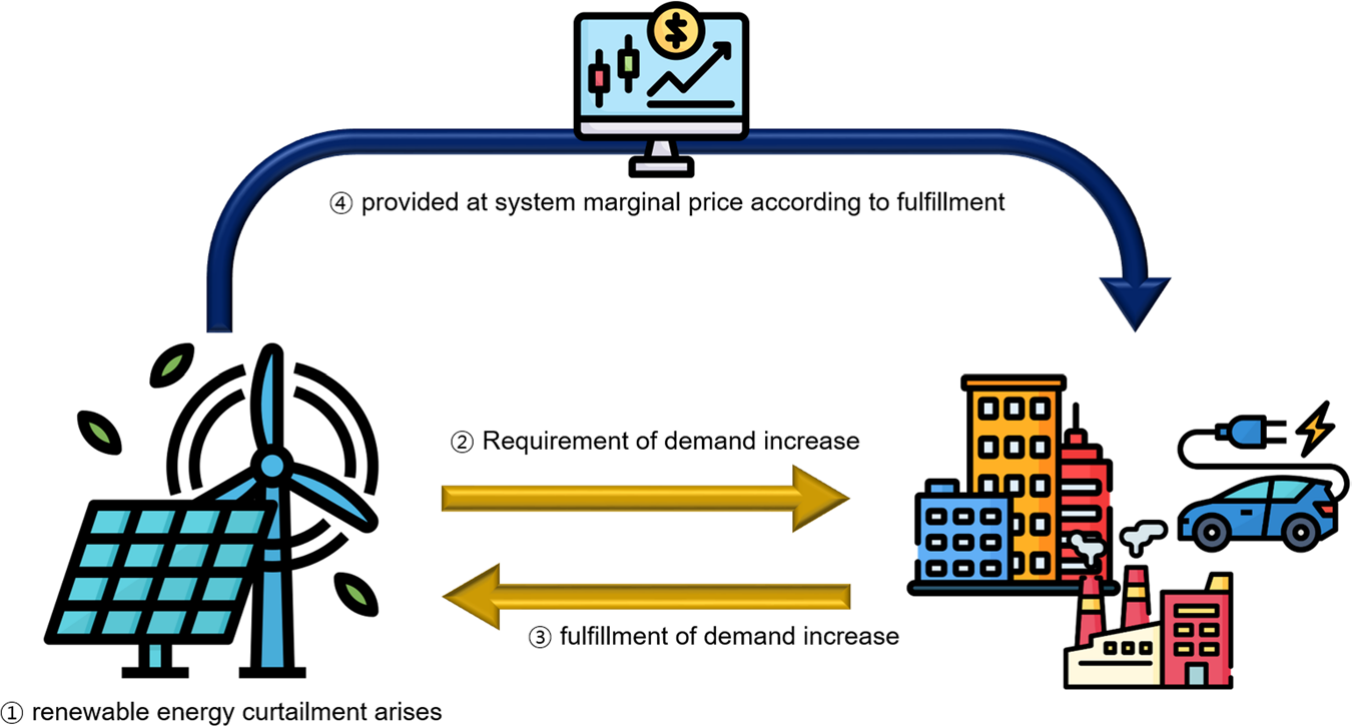
The process of plus DR.

Plus DR is a program introduced to reduce renewable energy curtailment while contributing to system stabilization. If the curtailment is expected, market operators request a demand increment, power consumers voluntarily increase their electricity usage, and renewable energy operators purchase electricity equivalent to excess power generation as shown in Fig. 3.

In order for microgrids to operate independently with renewable energy sources, a reverse DR market, called Plus DR program in South Korea, is being developed that can handle the surplus power generation of renewable energy. For the purpose of participating in the program, the characteristics of EVs and EV chargers in a general transaction environment should be studied from multiple perspectives. Therefore, in this study, data were investigated to estimate the potential for EV participation in the Plus DR program in South Korea.

## Data Records

As summarised in Tables 5, 6, the entire dataset consists of 2,337 EV users, 2,119 chargers, and 72,856 sessions^37^. The dataset is provided in the form of a comma-separated-value (CSV) file. In particular, EV users with IDs recorded as 0 refer to customers in this study who are not subscribed to a commercially operated company. Since the method of preprocessing data is selected and applied according to various research purposes, the authors provided raw data without preprocessing for reuse. As shown in Table 7, the dataset has 16 columns, including ChargingsessionID, UserID, ChargerID, ChargerCompany, Location, ChargerType, ChargerCapacity, ChargerACDC, StartDay, StartTime, EndDay, EndTime, SrartDatetime, EndDatetime, Duration, and Demand. The number of rows corresponds to the number of independent sessions. The data comprise all charging sessions that occurred during commercial operations from September 30, 2021, to September 30, 2022. The dataset has been made publicly available under the creative commons license CC BY 4.0 posted on the figshare repository.

**Table 5:** Summary of EV chargers (the number of chargers and sessions).

Charger	The number of chargers	The number of sessions
Own company	289	45713
Other company	1830	27143
Total	2119	72856

**Table 6:** Summary of EV chargers by location (the number of chargers and sessions).

Charger location	The number of chargers	The number of sessions
Apartment	281	14038
Accommodation	2	187
Hotel	133	8600
Resort	138	8854
Camping	8	1630
Market	106	3005
Restaurant	117	2222
Bus garage	7	29
Public parking lot	205	3396
Public area	437	14082
Public institution	402	7023
Company	141	7558
Sightseeing	118	1203
Golf CC	24	1029

**Table 7:** Summary of EV sessions dataset file.

Column	Description
ChargingsessionID	Charging Session ID
UserID	User ID; own members (1–2337) and other company’s members/non-members (0)
ChargerID	Charger ID
ChargerCompany	Categorization by charger company’s type: own company (1), other company (0)
Location	Installed location of charger; location type(accommodation, apartment, bus garage, camping, company, golf, hotel, market, public area, public institution, public parking lot, resort, restaurant, and sightseeing)
ChargerType	Categorization by charging speed; fast charger (1) and slow charger (0)
ChargerCapacity	Categorization by charging capacity
ChargerACDC	Categorization by charging type (i.e. AC and DC)
StartDay	Start date of connection between EV and charger (YYYY-MM-DD)
StartTime	Start time of connection between EV and charger (HH:MM:SS)
EndDay	End date of connection between EV and charger (YYYY-MM-DD)
EndTime	End time of connection between EV and charger (HH:MM:SS)
StartDatetime	Start date time of connection between EV and charger (YYYY-MM-DD HH:MM:SS)
EndDatetime	End date time of connection between EV and charger (YYYY-MM-DD HH:MM:SS)
Duration	Charger connection duration (unit: minute)
Demand	Amount of power charged to the EV (unit: kWh)

## Technical Validation

### Missing data

While communication failures can cause entire session events to be lost, no communication failures occurred during this study.

### Charger-side coupling statistics

**Fig. 4:**
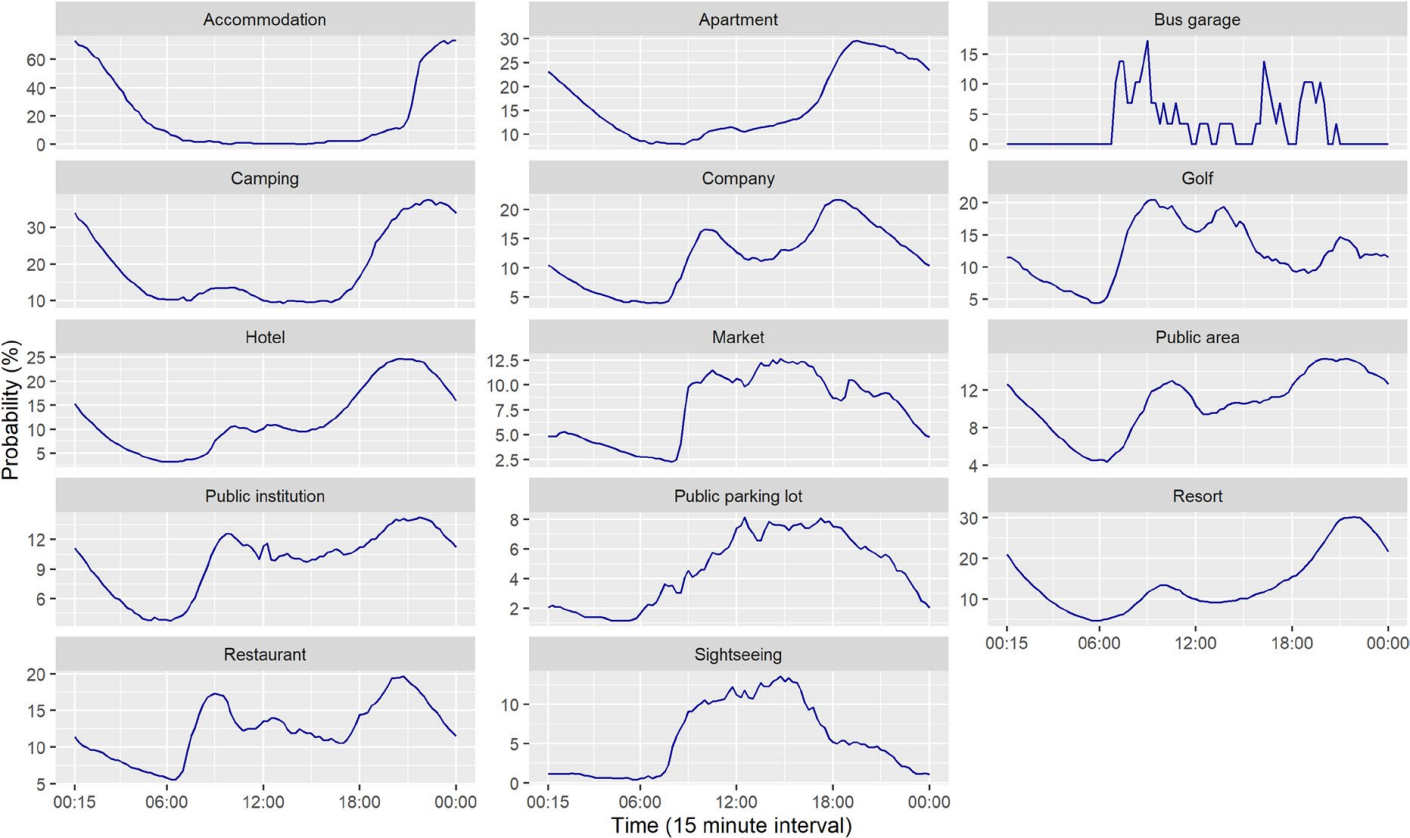
The daily coupling probability for each installation location.

**Fig. 5:**
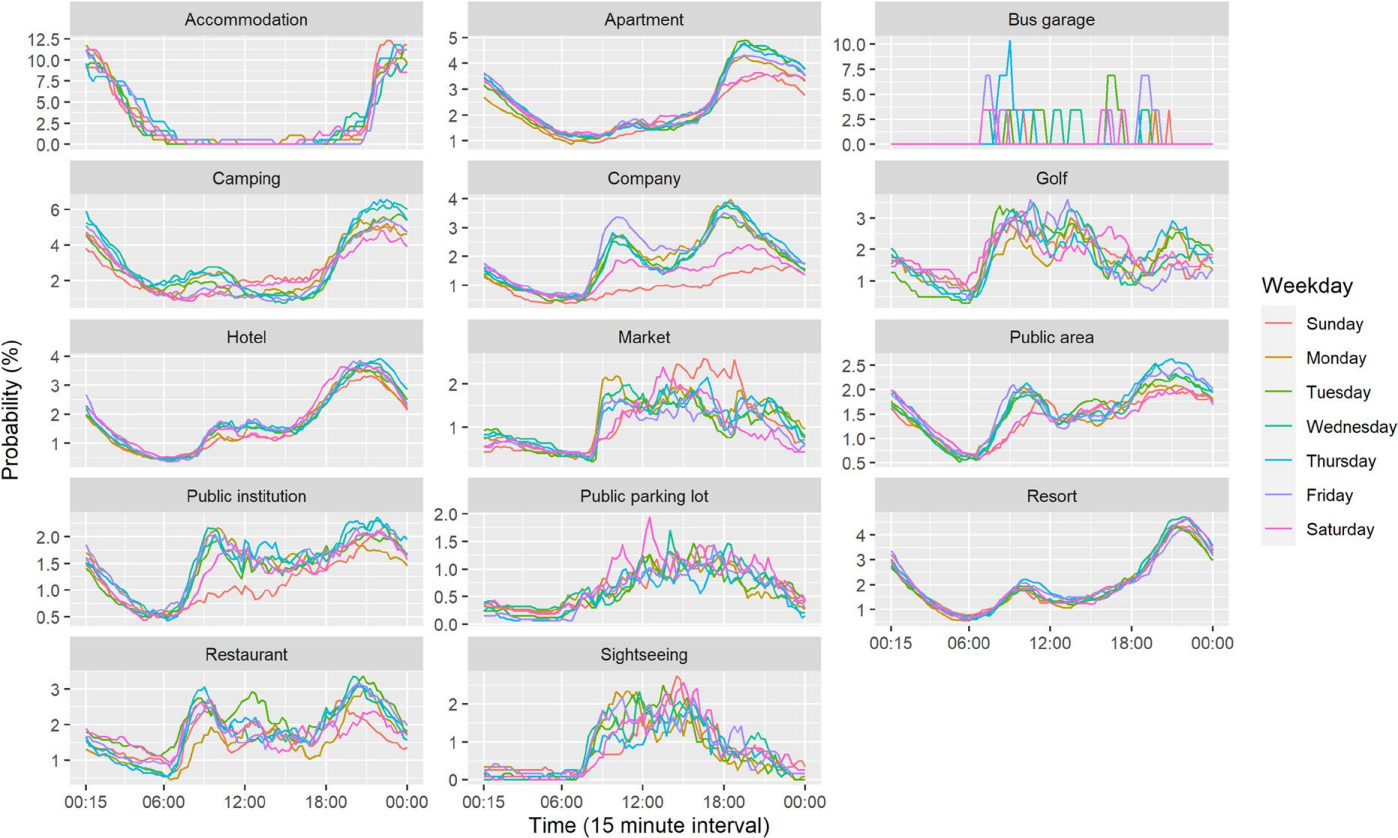
The daily coupling probability of each installation location for each day of the week.

The daily coupling probability for each installation location is estimated as shown in Fig. 4. Chargers installed in residential locations, such as accommodation, apartment, hotel, camping, and resort tend to charge in the evening and later hours. Other locations tend to have charging behaviour during the daytime. As shown in Fig. 5, the patterns for each day of the week are quite similar, with the exception of bus garages, which have a sporadic charging pattern. In addition, company, public institution, and apartment have weekday-weekend patterns. Table 8 shows the statistics of charger usage. On average, the operation rate and charger coupling duration over research periods are 41% and 3 days, respectively.

**Table 8:** Summary of charger usage statistics.

	Maximum	Median	Mean	Minimum
Operation rate	1	0.35	0.41	0.02
Average coupling duration	6.45	2.73	2.89	0.70

### User-side coupling statistics

**Fig. 6:**
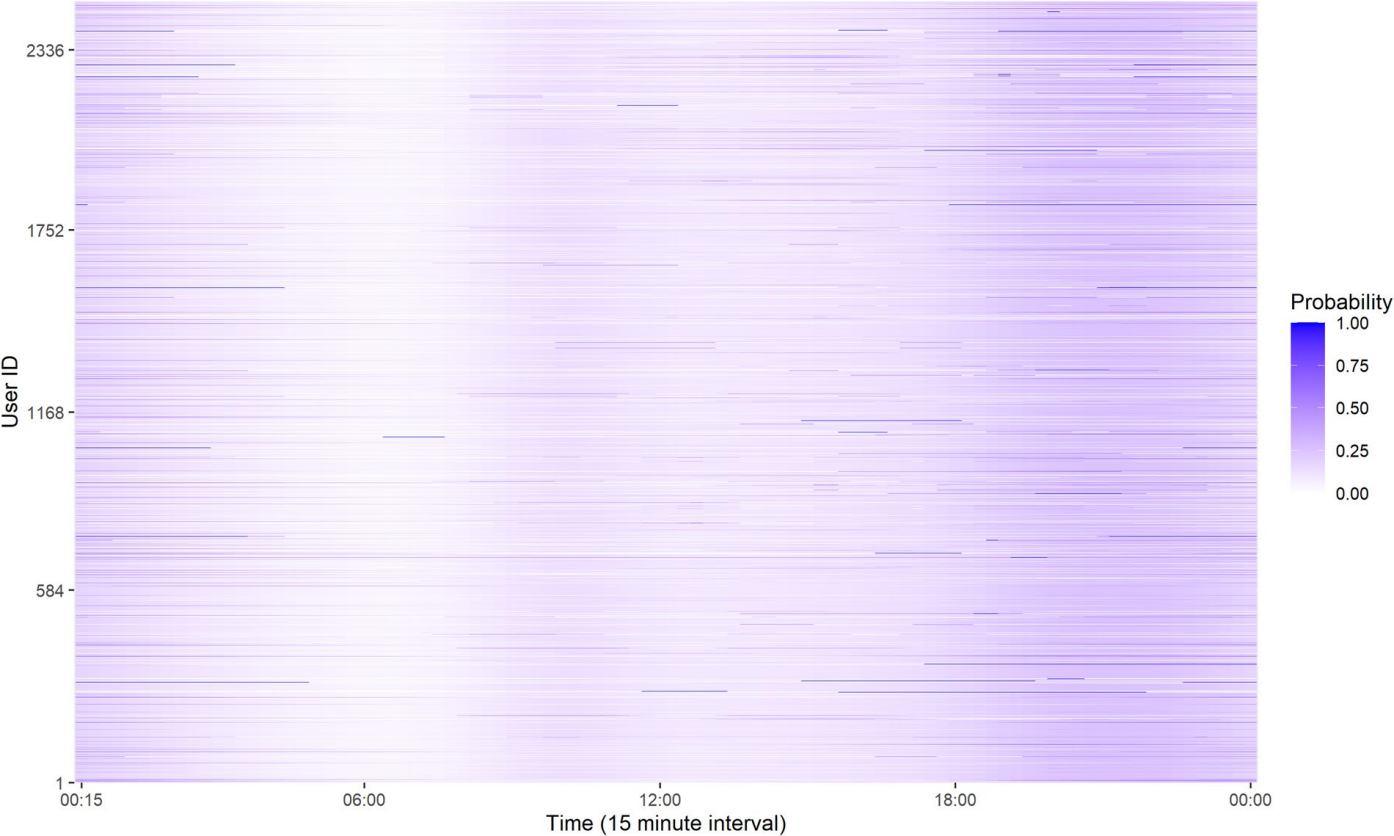
The heatmap of average daily coupling probability patterns for EV users.

**Fig. 7:**
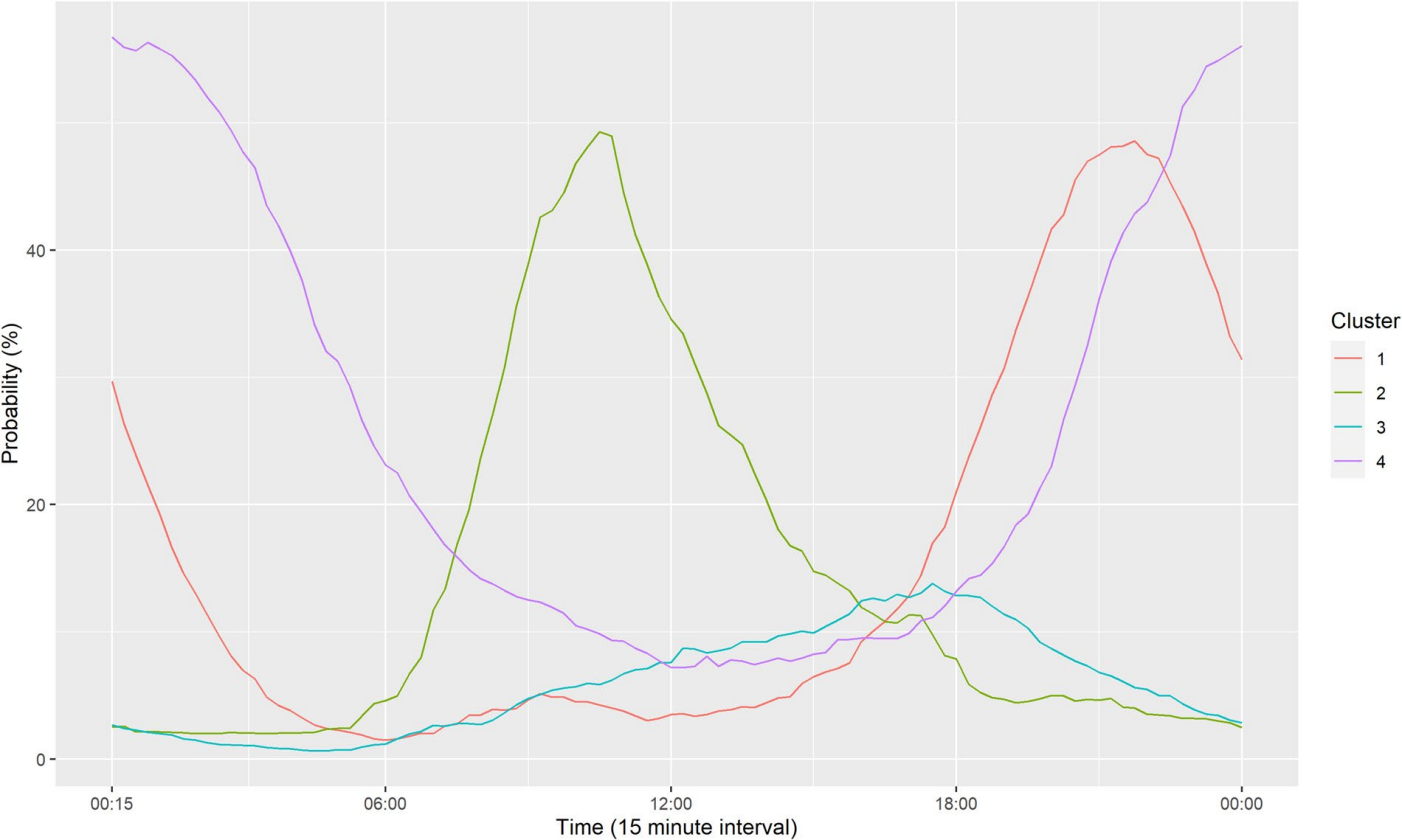
The representative daily coupling probability patterns for EV users.

**Fig. 8:**
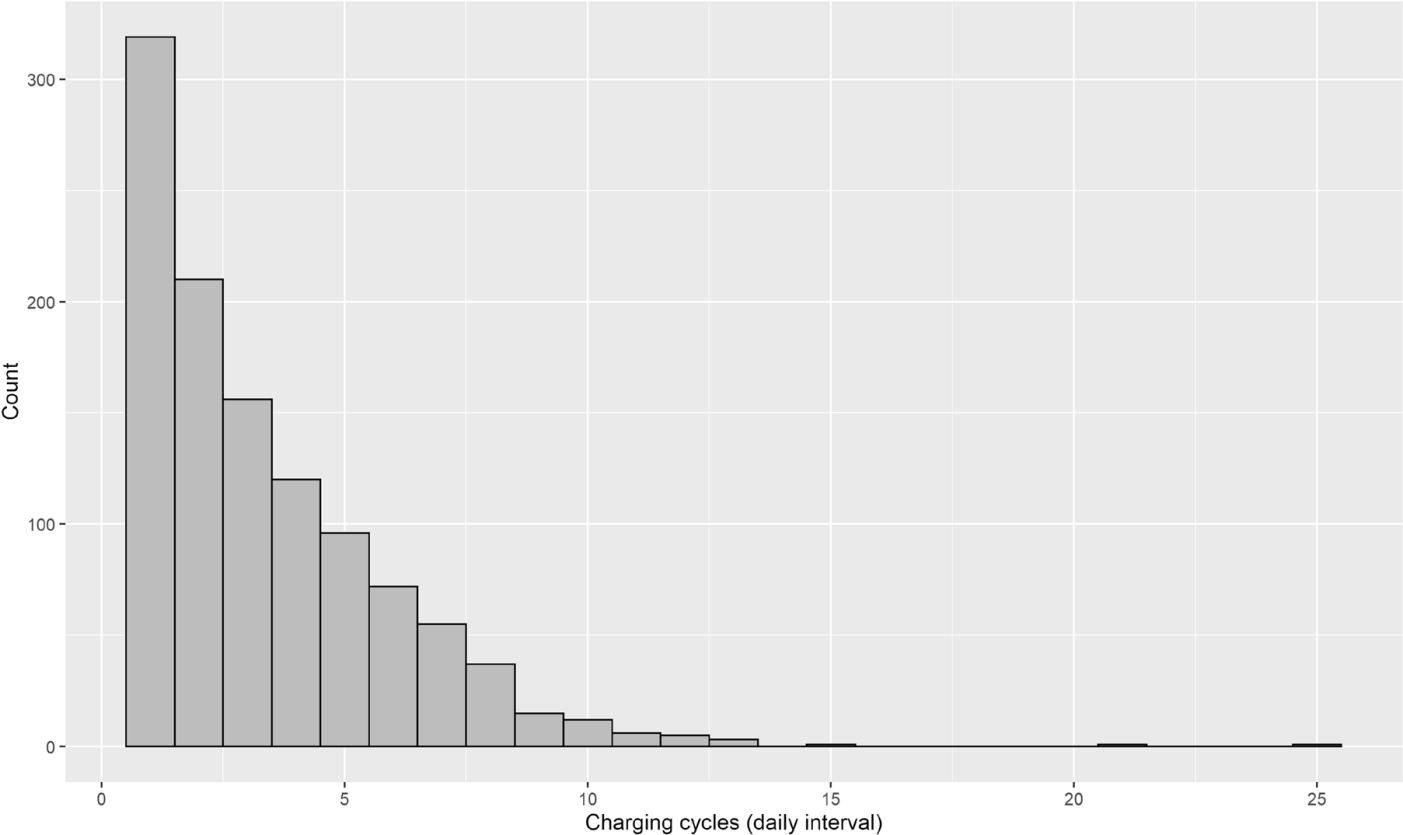
The histogram of daily average charging cycles for EV users.

**Fig. 9:**
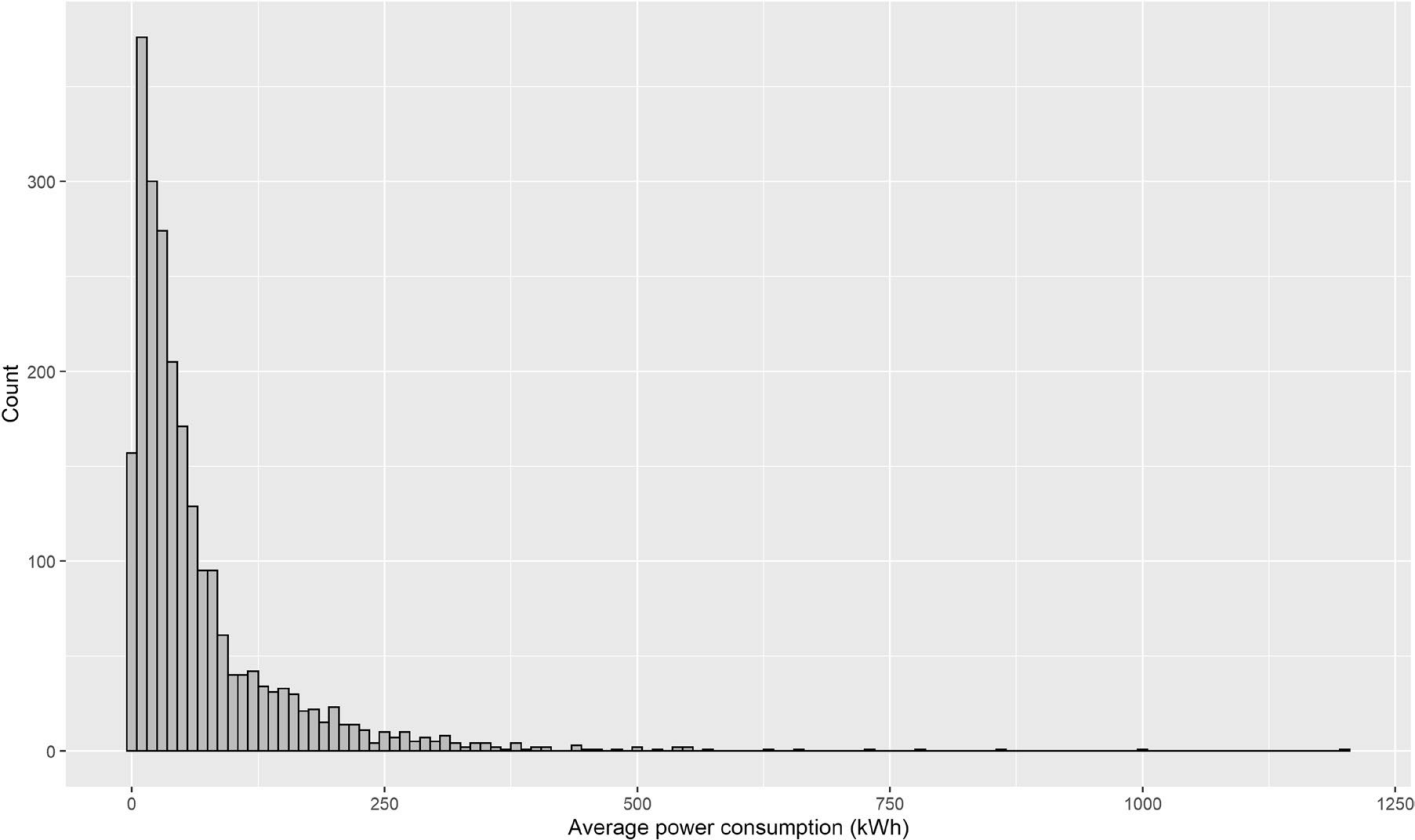
The histogram of monthly average power consumption for EV users.

Figure 6 shows the average coupling probability of users. The coupling behaviours of 2,337 charging platform subscribers out of the total users are estimated. The characteristics of major users are confirmed by decoupling in the early morning and coupling in the evening. To confirm the representative behavioural characteristics of users, a clustering methodology should be applied such as k-means, self-organizing map, fuzzy clustering, and Markov chain algorithms^38^. Based on K-means clustering into four groups, as shown in Fig. 7, EV users generally tend to start charging in the evening. Furthermore, the pattern consists of brief charging in the evening or at night, overnight charging, and aperiodic charging. As presented in Table 9 and Fig. 8, the average charging cycle is 3.43 days, and EV users with a charging cycle of one week or less account for 90.71% of the total. The remaining 1,072 users, representing 9.29%, are considered outliers and removed from the analysis. The monthly demand of the users is estimated as described in Table 10 and Fig. 9, with an average charging power consumption of 66.52KW.

**Table 9:** Summary of EV sessions dataset statistics (EV charging periods).

Statistics	Periods (days)
Max	25
Mean	3.43
Median	2.73
Minimum	1

**Table 10:** Summary of EV sessions dataset statistics (Monthly demand).

Statistics	Monthly demand (kWh)
Max	1202.75
Mean	66.52
Median	37.39
Minimum	0.01

### Arrival and departure time interval statistics

**Fig. 10:**
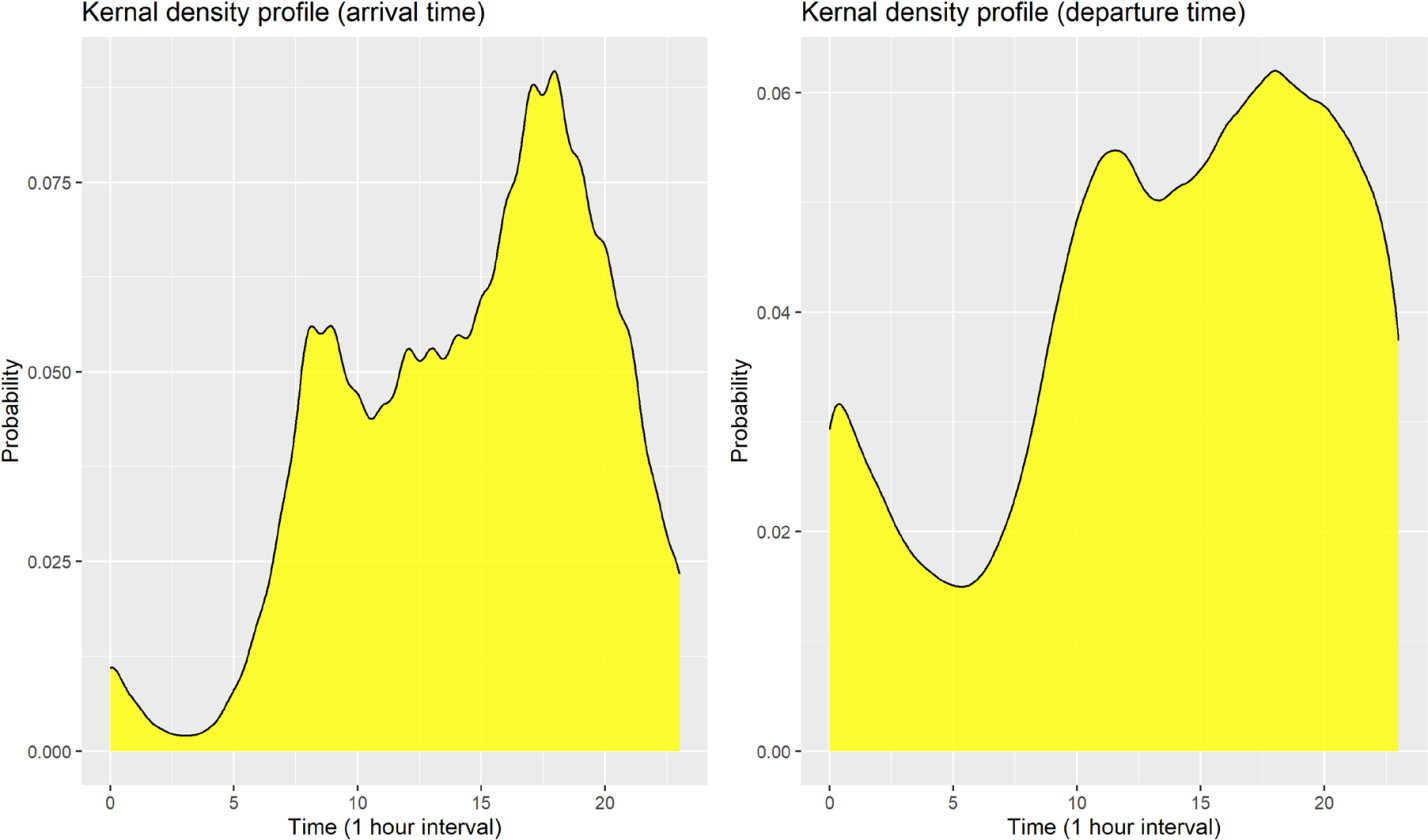
The kernel density profiles of arrival and departure times for charging sessions.

**Fig. 11:**
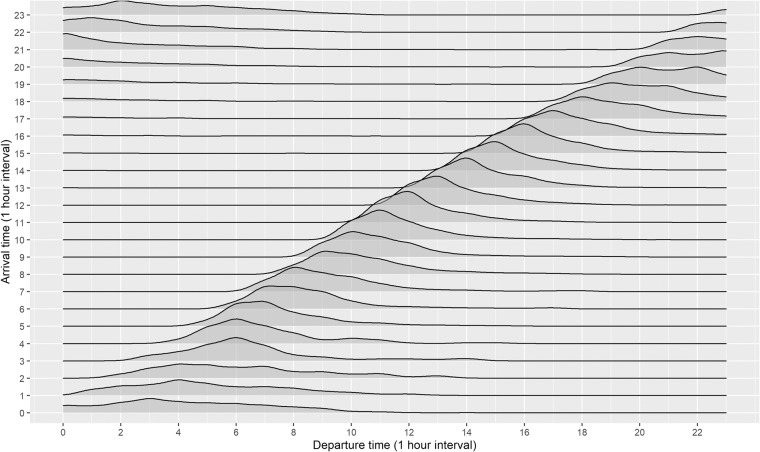
The probability density function for each arrival time according to departure times for charging sessions.

As shown in Fig. 10, a pattern of spikes in arrival times is confirmed mostly in the morning or evening. The kernel density profile for departure times is similar in shape to the profile of arrival times but was shifted several hours later. The probability density function of departure times for each arrival time is estimated as shown in Fig. 11. During the daytime (05:00–17:00), it can be confirmed that customers generally leave immediately after charging is complete. On the other hand, EVs arriving during the evening (18:00) have a pattern of remaining idle even after charging is complete.

## Data Availability

The code implementation was done in R 4.1.2 using R studio. The scripts to perform data visualization are available in^37^.
